# Intensive Care Unit Capacity in Low-Income Countries: A Systematic Review

**DOI:** 10.1371/journal.pone.0116949

**Published:** 2015-01-24

**Authors:** Srinivas Murthy, Aleksandra Leligdowicz, Neill K. J. Adhikari

**Affiliations:** 1 Department of Pediatrics, University of British Columbia, Vancouver, BC, Canada; 2 Interdepartmental Division of Critical Care, University of Toronto, Toronto, ON, Canada; 3 Department of Critical Care Medicine, Sunnybrook Health Sciences Centre, Toronto, Toronto, ON, Canada; Hospital Sirio-Libanes, BRAZIL

## Abstract

**Purpose:**

Access to critical care is a crucial component of healthcare systems. In low-income countries, the burden of critical illness is substantial, but the capacity to provide care for critically ill patients in intensive care units (ICUs) is unknown. Our aim was to systematically review the published literature to estimate the current ICU capacity in low-income countries.

**Methods:**

We searched 11 databases and included studies of any design, published 2004-August 2014, with data on ICU capacity for pediatric and adult patients in 36 low-income countries (as defined by World Bank criteria; population 850 million). Neonatal, temporary, and military ICUs were excluded. We extracted data on ICU bed numbers, capacity for mechanical ventilation, and information about the hospital, including referral population size, public accessibility, and the source of funding. Analyses were descriptive.

**Results:**

Of 1,759 citations, 43 studies from 15 low-income countries met inclusion criteria. They described 36 individual ICUs in 31 cities, of which 16 had population greater than 500,000, and 14 were capital cities. The median annual ICU admission rate was 401 (IQR 234-711; 24 ICUs with data) and median ICU size was 8 beds (IQR 5-10; 32 ICUs with data). The mean ratio of adult and pediatric ICU beds to hospital beds was 1.5% (SD 0.9%; 15 hospitals with data). Nepal and Uganda, the only countries with national ICU bed data, had 16.7 and 1.0 ICU beds per million population, respectively. National data from other countries were not available.

**Conclusions:**

Low-income countries lack ICU beds, and more than 50% of these countries lack any published data on ICU capacity. Most ICUs in low-income countries are located in large referral hospitals in cities. A central database of ICU resources is required to evaluate health system performance, both within and between countries, and may help to develop related health policy.

## Introduction

Providing acute care to critically ill patients is a global enterprise, regardless of health system capacity [1,2]. However, the high cost of trained healthcare workers, infrastructure, and supplies has limited the development of intensive care units (ICUs) in low-income countries [3]. Additionally, the effectiveness of traditionally resource-intensive critical care in such settings is unknown, and ICU expansion in areas of severe resource constraint is therefore controversial [4].

The burden of critical illness in low-income countries is large and likely to increase with growing urbanization, emerging epidemics and access to hospitals [5–7]. Therefore, data on critical care capacity, considering access to both physical resources and health care professionals, are essential for health system planning but generally lacking or difficult to find [8]. Our objectives were to systematically review the published literature on critical care capacity in low-income countries and to compare population-based estimates of ICU bed capacity to high-and middle-income countries where available.

## Methods

### Search strategy

We used broad search terms to capture all relevant studies reporting on the existence and characteristics of ICUs, as defined by study authors, in target countries (see S1 File). Target countries were defined by the World Bank as low-income, corresponding to a 2012 gross national income per capita of less than USD1,035 [9].

With the assistance of a librarian, we searched 11 databases, including Medline, EMBASE, LILACS, African Index Medicus, African Journals Online, African Healthline, Opengrey, MedCarib, IMEMR, IMSEAR, and WPRIM. We developed a comprehensive search strategy based on commonly used critical care terms, using keyword and controlled vocabulary terminologies. We searched studies from January 1, 2004—August 6, 2014 with no language restrictions (see S1 File for search strategies). We restricted the search to the past 10 years, reasoning that older studies may underestimate current capacity and would therefore be less useful for health system planning.

We included studies of any design that included information about pediatric and adult ICU capacity (number of beds) in our pre-specified list of countries. Reviews and editorials were included only if they provided new data. We excluded studies reporting only neonatal ICU data and those that focused on temporary or military hospitals.

### Searching and data abstraction

Two reviewers (SM, AL) independently screened titles, citations, and abstracts for potentially relevant studies. Full-text versions of all potentially eligible studies were retrieved and reviewed by the same reviewers for inclusion in the review. Agreement between the two reviewers for inclusion of studies among full-text articles was measured using κ [10]. Disagreements were resolved by consensus and adjudication by a third reviewer (NA). Two data abstractors (SM, AL) independently extracted data from selected studies, including demographic data of treated patients, hospital and ICU bed numbers, mechanical ventilation capacity, referral population size, public accessibility, and the hospital funding. If more than one study discussed the same ICU, we abstracted data from the more recent study. Where required, we obtained translations of non-English studies. For studies reporting on national ICU capacity, we extracted total national hospital bed data from World Bank databases [9].

### Statistical analysis

We summarized continuous data as mean (standard deviation, SD) if normally distributed or median (interquartile range, IQR) if not normally distributed, and categorical data as count (percentage). We constructed plots and created regression lines of ICU beds per population vs. hospital beds per population using data from included studies and reviews of ICU capacity in high-income countries [11] and plots of ICU beds per population vs. national healthcare expenditure per population using World Bank data [9]. All statistical analyses and plots were performed using R version 3.1.1(R Project for Statistical Computing, Vienna Austria).

## Results

**Figure 1:**
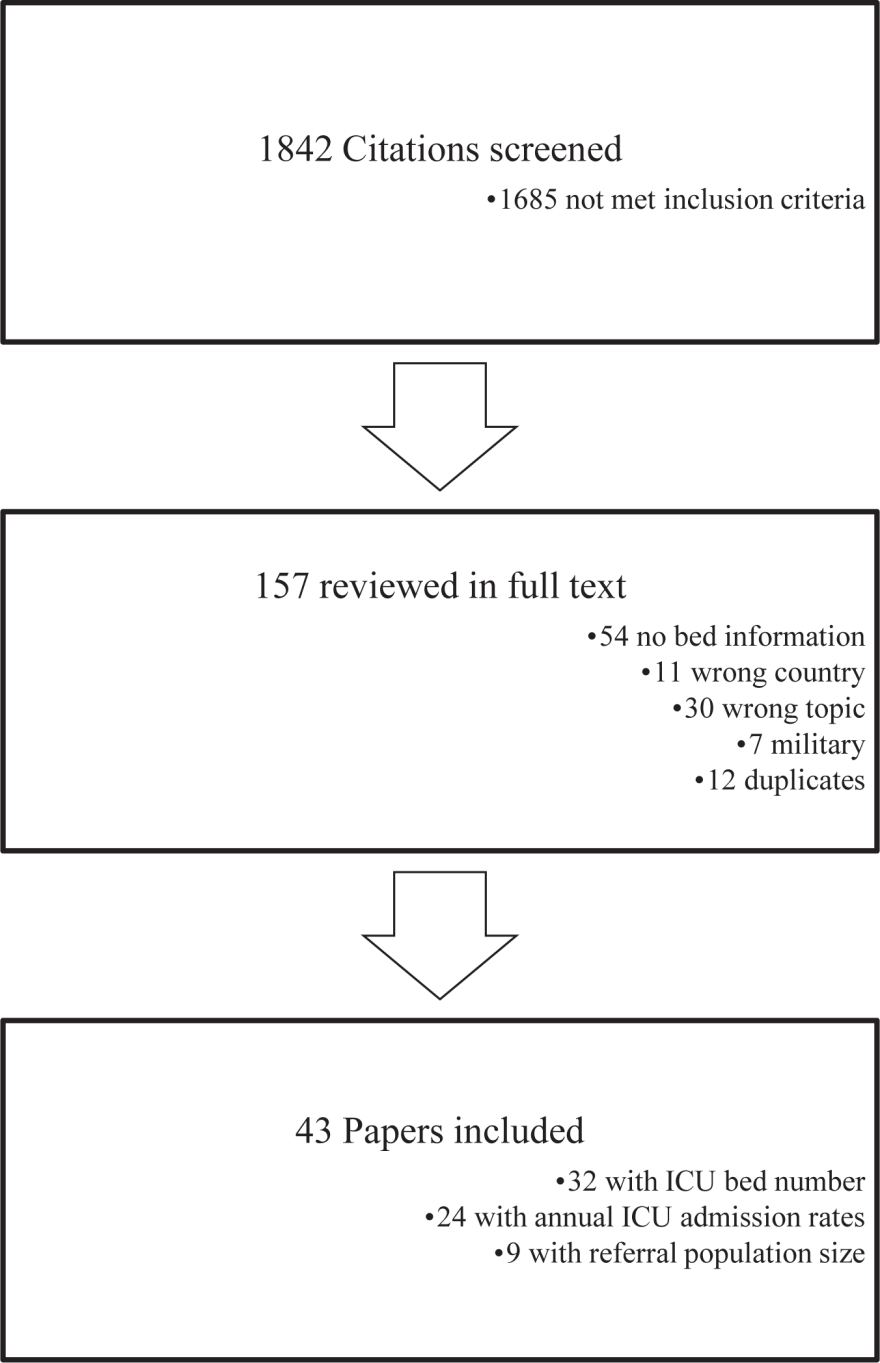
Flow diagram of study selection. References for the citations excluded after full-text review are provided in S1 File.

Our search identified 1759 articles, 1603 of which did not meet inclusion criteria after title and abstract review (Fig. 1). Of the 153 articles selected for full-text review, 110 were excluded (see S1 File), with 43 articles meeting selection criteria for final analysis (κ = 0.72, 95% confidence interval, 0.60–0.83). These studies described 36 individual ICUs (Table 1). Four included studies were published in abstract form only and 13 were identified only through searching journals not available on MEDLINE or EMBASE.

**Table 1:** Details of ICU capacity in low-income countries from published studies.

Country	City	Hospital	ICU type	Hospital bed number^a^	ICU bed number^a^	Annual ICU admissions^a^	Referral Population	Reference
**Cambodia**	Phnom-Penh	Cardiological Center of Phnom-Penh	Cardiac, Adult	32	8	No data	No data	[28]
**Cambodia**	Siem Reap	Angkor Hospital for Children	Pediatric	50	4	725	No data	[29]
**Comoros Islands**	El Maarouf	Centre Hospitalier Regional	Adult	No data	10	760	No data	[30]
**Democratic Republic of Congo**	Lubumbashi	Provincial Hospital Jason Sendwe	No data	No data	No data	257	No data	[31]
**Democratic Republic of Congo**	Goma	DOCS Hospital	Adult	No data	3	141	No data	[32]
**Eritrea**	Asmara	Orotta national Referral Hospital	Adult	300	9	390	No data	[33]
**Ethiopia**	Addis Ababa	Yekatit 12 Hospital	Burn unit Adult & Pediatric	No data	18	No data	No data	[34]
**Ethiopia**	Addis Ababa	Black Lion Hospital	No data	No data	No data	276	No data	[35]
**Ethiopia**	Addis Ababa	Tikur Anbassa Hospital	Adult, Medical	500	6	591	No data	[36]
**Ethiopia**	Jimma	Jimma University Specialized Hospital	Adult	450	6	370	15 million	[37,38]
**Kenya**	Kilifi	Kilifi District Hospital	Pediatric	60	No data	No data	200 000	[39]
**Kenya**	Nairobi	Kenyatta National Hospital	Adult, Pediatric	1800	20	1200	32 million	[40]
**Kenya**	Nairobi	Mater Hospital	Adult	140	5	No data	No data	[41]
**Kenya**	Nakuru	Nakuru Provincial Hospital	No data	750	5	No data	No data	[42]
**Malawi**	Blantyre	Queen Elizabeth Central Hospital	Adult	No Data	4–5	No data	No data	[43]
**Malawi**	Lilongwe	Kamuzu Central Hospital	Adult	600	4	234	9 million	[15]
**Mali**	Bamako	CHU Gabriel Toure	No data	No Data	No data	555	No data	[44]
**Nepal**	Dharan	Koirala Institute of Health Science	Pediatric	No data	6 (Adult)	425 (Adult)	No data	[45,46]
**Nepal**	Dhulikhel	Dhulikhel Hospital	Adult	340	5	No data	No data	[47]
**Nepal**	Kathmandu	Tribhuban University Teaching Hospital	Adult, Pediatric	No data	6	234	No data	[48,49]
**Nepal**	Mix	Mix	Adult, Pediatric (48 total)	7040 (whole country)	450, 60	No data	29 million	[18]
**Nepal**	Patan	Patan Hospital	Pediatric	No data	16, 6	126 (pediatric)	No data	[50–52]
**Nepal**	Pokhara	Manipal Teaching Hospital	Adult, Pediatric	750	11	992*	3 million	[53,54]
**Nepal**	Thapathali	Norvic International Hospital and Medical Center	Adult	No data	No data	700	No data	[55]
**Niger**	Mirriah town	Mirriah District Hospital	Pediatric	No data	10	No data	No data	[56]
**Tanzania**	Dar es Salaam	Muhimbili National Hospital	Adult	1000	10	412	No data	[57,58]
**Tanzania**	Ifakara	St. Francis Hospital	No Data	No data	10	715	No data	[32]
**Tanzania**	Mwanza	Sekou Toure Regional Referral Hospital	Adult	375	8	No data	3.2 million	[59]
**Tanzania**	Mwanza	Budago Medical Center	Adult, Pediatric	1000	12, 10	No data	13 million	[16]
**Togo**	Lome	Tokoin University Hospital Center	No data	No data	No data	1689	No data	[60]
**Uganda**	Gulu	St. Mary's Hospital Lacor	Adult, Pediatric	476	8	218	No data	[61,62]
**Uganda**	Kampala	Mulago Hospital	Adult, Pediatric, Cardiac	1500	12, 6, 4	222 (Adult)	No data	[14,63]
**Uganda**	Masaka	Masaka Regional Referral Hospital	Adult	No data	1	No data	No data	[63]
**Uganda**	Mbarara	Mbarara Hospital	Adult, Pediatric	No data	2 (increasing to 8)	No data	3 million	[23,64]
**Zambia**	Lusaka	University Teaching Hospital	No data	1300	5	No data	No data	[42]
**Zimbabwe**	Harare	Parirenyatwa Hospital	Pediatric	No data	5	102	No data	[65]

^a^Where more than one reference was available for the same hospital, we used the most recent reference for hospital, ICU bed numbers, and ICU admissions per year.

**Figure 2:**
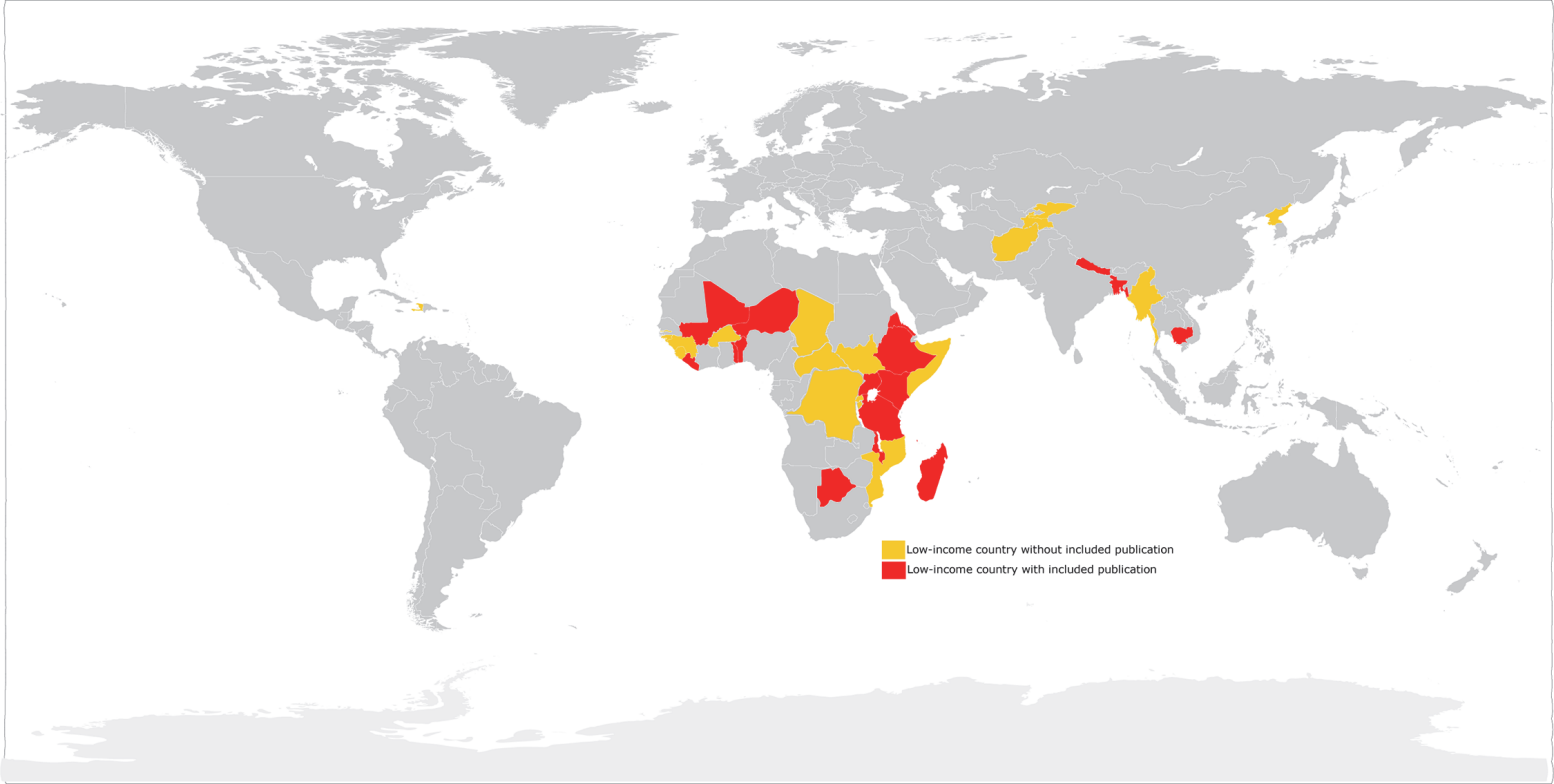
Thirty-six low-income countries included in the search strategy with (n = 15, red) and without (n = 21, yellow) published data on ICU resource availability.

The articles included described ICU capacity in a pre-specified hospital (n = 40) or national (n = 3) region. Based on listed affiliations, 40% (17/43) of articles had corresponding authors based in the low-income country. Of 36 low-income countries defined by the World Bank, only 15 (42%) countries had studies meeting our inclusion criteria (Fig. 2). Therefore, most low-income countries had no literature on ICU capacity, or described ICUs that did not meet our inclusion criteria, such as relief or military hospitals [12,13].

The definitions of critical care varied across the few studies that provided explicit definitions. One described ICU beds as requiring a 'pulse oximeter, mechanical ventilator, suction machine and an anesthesia provider'[14]. Others described the ICU as a 'specialized unit with more skilled nursing care,'[15] or a 'concentrated area where the level of care and supervision is considerably more sophisticated than in the ordinary ward'[16].

**Figure 3:**
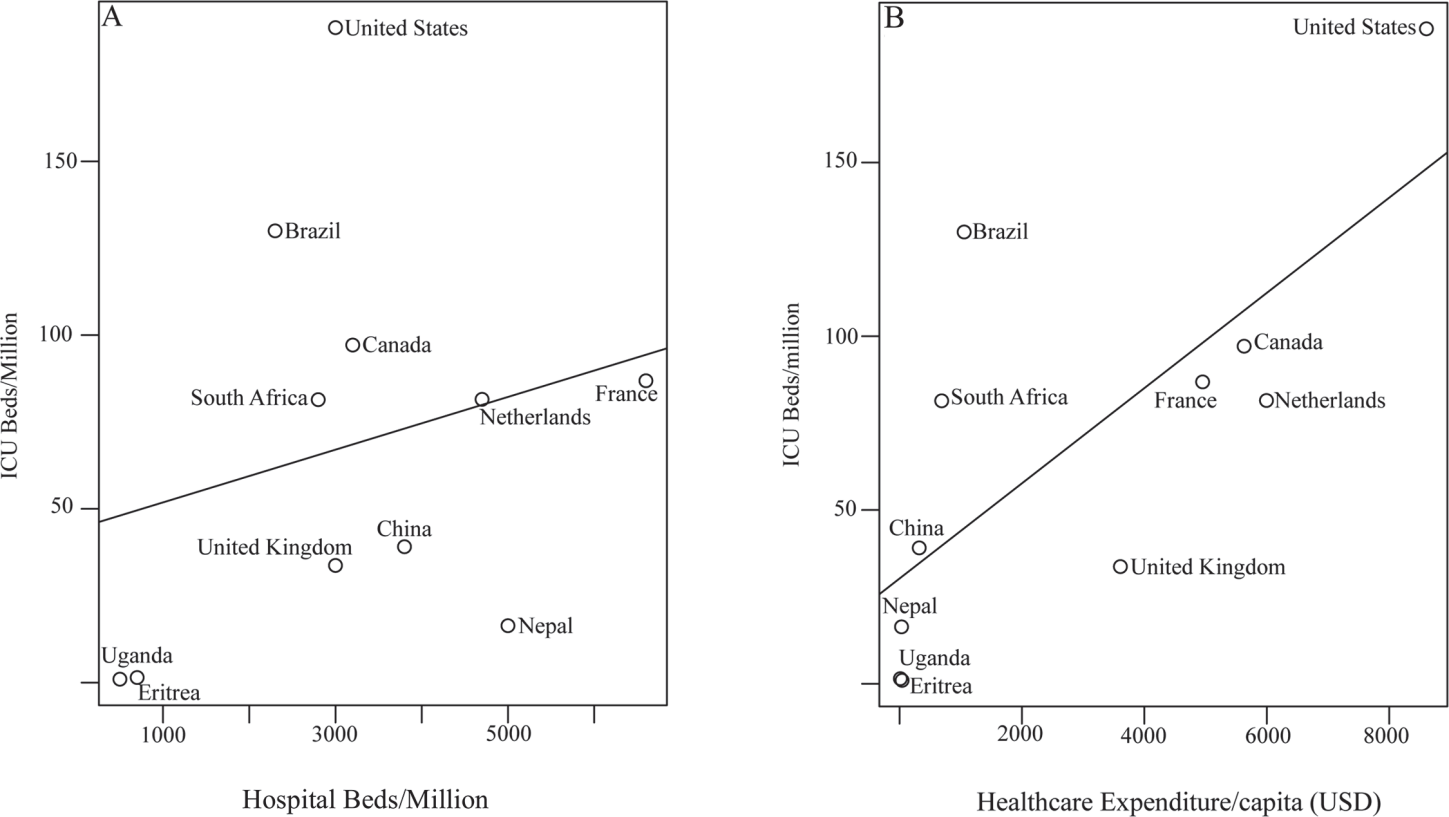
Comparison of the relationship between ICU beds and hospital beds (panel a), and between ICU beds and national healthcare expenditure per capita (panel b) in low versus selected high-income countries. There is a non-significant trend between ICU beds and hospital beds (R^2^ = 0.11, p = 0.37; R^2^ = 0.24, p = 0.12 if USA is excluded) and a significant trend between ICU beds and national healthcare expenditure per capita (R^2^ = 0.76, p = 0.002). Supplementary data are from [26,27].

The 43 studies collectively described 36 individual ICUs. Only 3 studies explicitly quantified ICU capacity across a geographic region [17] or country [14,18]; the remaining 40 studies provided single-center descriptions (Table 1). Nine of these 40 studies provided details on referral population size without stating whether additional ICU capacity existed in other hospitals serving the same catchment area. The 36 individual ICUs were distributed among 31 cities, of which 16 had a population greater than 500,000 and 14 were national capitals. Most ICUs (94.1%, 32/34 with data on hospital type) were located in large referral hospitals in major cities. Nepal and Uganda, the only countries with national ICU bed data, had 16.7 and 1.0 ICU beds per million population, respectively [14,18]. When comparing national critical care capacity among low-income countries with data from this review and other countries from other sources [refs], the number of ICU beds per population (Fig. 3) is poorly associated with the number of hospital beds per population (R^2^ = 0.11, p = 0.37; R^2^ = 0.24, p = 0.12 if USA is excluded) and strongly associated with annual national healthcare expenditure per capita (R^2^ = 0.75, p = 0.002).

The median annual number of ICU admissions was 401 (IQR 234–711; 24 ICUs with data) the median ICU size was 8 beds (IQR 5–10; 32 ICUs with data), and the median annual admission rate per ICU bed was 58.5 (IQR 41–71, 13 ICUs with data). The mean number of adult and pediatric ICU beds, as a percentage of hospital beds, was 1.5% (SD 0.9%; 15 hospitals with data). Thirteen (36.1%) ICUs explicitly mentioned accepting pediatric patients.

Twenty-two (61%) ICUs provided data on mechanical ventilation capacity, of which 17 (77%) had mechanical ventilators. No study reported whether ICU access was privately or publicly funded. There were few data on physician staffing (1 of 36 ICUs), nurse:patient ratios (2 of 36 ICUs), or the presence of an educational mandate (10 of 36 ICUs).

## Discussion

Based on currently available literature, access to critical care resources in World Bank-defined low-income countries is poorly described on the level of individual ICUs and even more sparingly described on a population level. By reported measures, however, ICU resources in low-income countries appear to be sparse. Not surprisingly, the number of ICU beds nationally was related to overall hospital bed capacity and even more significantly to the national expenditure on healthcare.

The inconsistency of definition of an ICU bed implies a significant challenge when comparing resources among countries; even within high-income countries, the definition of an ICU may depend on a higher nurse:patient ratio, the availability of mechanical ventilation, or the ability to support multiple organ systems simultaneously[11,19]. Future research must acknowledge these differences when performing international comparisons [20]. A consensus definition of an ICU, stratified by overall healthcare system capacity, would help with standardizing data collection and may help with planning evaluations of interventions to improve the care and outcomes of seriously ill hospitalized patients.

It is important to note that the mere presence of an ICU does not imply the ability to effectively care for critically ill patients. Similarly, counting the number of critically ill patients from the number of ICU beds in countries with insufficient capacity will lead to a gross underestimate [8]. As reported in a survey of African providers, the ability to comply with sepsis guidelines is minimal in most of Sub-Saharan Africa, despite the presence of an ICU [21]. Additionally, data on many of the features unique to critical care, such as mechanical ventilation and increased nurse:patient ratios, were absent in many of the ICUs described, belying their ability to provide care to critically ill patients. Therefore, there is an urgent need for cross-institutional collaboration for the collection of standardized resource and outcome data through registries and for sharing of appropriate management strategies in resource-constrained low-income countries [2].

There is negligible published research emerging from critical care communities in low-income countries [22]. Reasons for this may include the lack of critical care providers and researchers, funding, academic mentorship, infrastructure to perform research, or barriers to developing available data into publishable research. Given the high burden of critical illness in low-income regions with a collective population of 850 million, the high mortality for patients admitted to ICUs in these countries, and the availability of strategies for their management, there is a rationale for ICUs in all regions of the world [2,23,24]. This must be balanced, however, against the opportunity costs in healthcare systems facing broad challenges of insufficient finding, too few healthcare workers, and poor infrastructure [25]. These challenges notwithstanding, knowledge of pre-existing ICU capacity is vital to plan any future development. For example, in a recent observational study that aimed to assess the worldwide burden of critical illness through convenience sampling of ICU admissions, only 2 of the 84 countries were low-income and 2 of the 730 participating centers were from low-income countries [2].

This systematic review has a number of limitations. While our search strategies were exhaustive, we were unable to capture data on all ICUs in a region due to the lack of relevant publications. Searching of published research literature is an insensitive tool for resource determination, and centralized databases are required to estimate actual acute healthcare capacity [8]. Furthermore, there was substantial variability in availability of relevant data among the included studies and we often relied on single-sentence statements of critical care capacity. Additionally, no independent validation of results was performed. Many health systems have changed drastically since the publication of the studies included in this systematic review, and given the ten-year time frame of our data collection, some features of critical care described may be outdated.

## Conclusions

Reliable published data on the availability of critical care resources in low-income regions is sparse. Existing critical care resources are modest and positively associated with national hospital bed capacity and healthcare spending. A global database of ICU capacity, facilitated by networks of critical care providers in low-income countries, would help to evaluate access to and outcomes from critical care, both within and between countries.

## Supporting Information

S1 File Search Strategy, Country List, Reference list: Excluded papers. (DOCX)

S1 PRISMA Checklist. (DOC)
