# Supplementary material for: Intensive Care Unit Capacity in Low-Income Countries: A Systematic Review
Source: PLoS One. 2015 Jan 24;10(1):e0116949. doi: 10.1371/journal.pone.0116949 (PMC4305307; doi:10.1371/journal.pone.0116949)
Supplement: S1 File — (DOCX) [file pone.0116949.s001.docx]

**Title: Critical Care Capacity in Low-Income Countries: A Systematic Review**

Supplementary Appendix

**Search Strategy:**

--------------------------------------------------------------------------------

1 exp Intensive Care Units/ or exp Intensive Care/ or exp Critical Care/

2 afghanistan.cp,ab,in,sh.

3 bangladesh.cp,ab,in,sh.

4 benin.cp,ab,in,sh.

5 burkina faso.cp,ab,in,sh.

6 burundi.cp,ab,in,sh.

7 cambodia.cp,ab,in,sh.

8 central african republic.cp,ab,in,sh.

9 chad.cp,ab,in,sh.

10 comoros.cp,ab,in,sh.

11 Democratic Republic of the Congo.cp,ab,in,sh.

12 eritrea.cp,ab,in,sh.

13 ethiopia.cp,ab,in,sh.

14 gambia.cp,ab,in,sh.

15 guinea.cp,ab,in,sh.

16 Guinea-Bissau.cp,ab,in,sh.

17 haiti.cp,ab,in,sh.

18 kenya.cp,ab,in,sh.

19 north korea.cp,ab,in,sh.

20 kyrgyz republic.cp,ab,in,sh.

21 liberia.cp,ab,in,sh.

22 madagascar.cp,ab,in,sh.

23 malawi.cp,ab,in,sh.

24 mali.cp,ab,in,sh.

25 mozambique.cp,ab,in,sh.

26 myanmar.cp,ab,in,sh.

27 nepal.cp,ab,in,sh.

28 niger.cp,ab,in,sh.

29 rwanda.cp,ab,in,sh.

30 sierra leone.cp,ab,in,sh.

31 somalia.cp,ab,in,sh.

32 south sudan.cp,ab,in,sh.

33 tajikistan.cp,ab,in,sh.

34 tanzania.cp,ab,in,sh.

35 togo.cp,ab,in,sh.

36 uganda.cp,ab,in,sh.

37 zimbabwe.cp,ab,in,sh.

38 icu.mp.

39 intensive care.mp.

40 exp Respiration, Artificial/

41 or/2-37

42 1 or 38 or 39 or 40

43 41 and 42

44 limit 43 to yr="2004 -Current"

***************************

**Country List**

Afghanistan, Bangladesh, Benin, Burkina Faso, Burundi, Cambodia, Central African Republic, Chad, Comoros, Democratic Republic of Congo, Eritrea, Ethiopia, Gambia, Guinea, Guinea-Bissau, Haiti, Kenya, Democratic Republic of Korea, Kyrgyz Republic, Liberia, Madagascar, Malawi, Mali, Mozambique, Myanmar, Nepal, Niger, Rwanda, Sierra Leone, Somalia, South Sudan, Tajikisan, Tanzania, Togo, Uganda, Zimbabwe

**Papers reviewed and subsequently excluded^1-108^**

No Bed information

1. Acharya SP, Pradhan B, Marhatta MN. Application of "the Sequential Organ Failure Assessment (SOFA) score" in predicting outcome in ICU patients with SIRS. Kathmandu University medical journal 2007;5:475-83.

4. Agalu A. Medication errors and associated factors in the intensive care unit Jimma University specialized hospital in Ethiopia, April, 2011. 2011 Annual Meeting of the American College of Clinical Pharmacy; 2011; Pittsburgh, United States.

5. Aguemon AR, Padonou JL, Yevegnon SR, et al. [Intensive care management of patients with severe head traumatism in Benin from 1998 to 2002]. Annales francaises d'anesthesie et de reanimation 2005;24:36-9.

6. Amare A, Yami A. Case-fatality of adult tetanus at Jimma University Teaching Hospital, Southwest Ethiopia. African health sciences 2011;11:36-40.

7. Andrews WR. Healthcare in the developing world: is targeting HIV enough? The case for prioritizing critical care. Critical care medicine 2011;39:916-7.

11. Baelani I, Jochberger S, Laimer T, et al. Availability of critical care resources to treat patients with severe sepsis or septic shock in Africa: a self-reported, continent-wide survey of anaesthesia providers. Critical care 2011;15:R10.

12. Bajracharya GR, Goonasekera C, Ismail S. Producing a research culture in anesthesiology and intensive care in the SAARC region. Anaesthesia, Pain and Intensive Care 2012;16:4-6.

13. Baker T, Lugazia E, Eriksen J, Mwafongo V, Irestedt L, Konrad D. Emergency and critical care services in Tanzania: a survey of ten hospitals. BMC health services research 2013;13:140.

14. Basher A, Rahman MF, Faiz MA. Intensive caremanagement for tetanus-A critical need. 28th International Congress of Chemotherapy and Infection Incorporating the 14th Asia-Pacific Congress of Clinical Microbiology and Infection; 2013; Yokohama, Japan.

15. Bibi S, Chisti MJ, Akram F, Pietroni MA. Ampicillin and gentamicin are a useful first-line combination for the management of sepsis in under-five children at an urban hospital in Bangladesh. Journal of health, population, and nutrition 2012;30:487-90.

16. Bista DB, Saha A, Mishra P, Palaian S, Shankar PR. Pattern of potential drug-drug interactions in the Intensive Care Unit of a Teaching Hospital in Nepal: A pilot study. Journal of Clinical and Diagnostic Research 2009;3:1713-16.

19. Chandler H, Son M, Colimon J. Defining barriers in caring for seriously ill children in a resource-limited setting: An observational study of 2 children's hospitals in Haiti. 6th World Congress on Pediatric Critical Care; 2011; Sydney, Australia.

20. Chisti MJ, Duke T, Robertson CF, et al. Clinical predictors and outcome of hypoxaemia among under-five diarrhoeal children with or without pneumonia in an urban hospital, Dhaka, Bangladesh. Tropical Medicine and International Health 2012;17:106-11.

21. Chisti MJ, Pietroni MA, Smith JH, Bardhan PK, Salam MA. Predictors of death in under-five children with diarrhoea admitted to a critical care ward in an urban hospital in Bangladesh. Acta paediatrica 2011;100:e275-9.

22. Chisti MJ, Salam MA, Ashraf H, et al. Clinical risk factors of death from pneumonia in children with severe acute malnutrition in an urban critical care ward of Bangladesh. PloS one 2013;8:e73728.

25. Coulibaly Y, Diango DM, Goita D, et al. Prevalence and mortality of infections on the interdisciplinary Intensive Care Unit in Mali, Africa. 4th International Congress "Sepsis and Multiorgan Dysfunction; 2009; Weimar, Germany.

27. Dhungel S, Bista S. High blood glucose level and increased risk of mortality in critically ill patients. Nepal Medical College journal : NMCJ 2007;9:44-5.

28. Diango DM, Almeimoune AM, Beye SA, et al. [Problems related to informing families of patients in intensive care unit AT CHU Gabriel Toure (Mali)]. Le Mali medical 2012;27:66-70.

30. Embu HY, Yiltok SJ, Isamade ES, Nuhu SI, Oyeniran OO, Uba FA. Paediatric admissions and outcome in a general intensive care unit. African journal of paediatric surgery : AJPS 2011;8:57-61.

31. Firth P, Ttendo S. Intensive care in low-income countries--a critical need. The New England journal of medicine 2012;367:1974-6.

34. Godebo G, Kibru G, Tassew H. Multidrug-resistant bacterial isolates in infected wounds at Jimma University Specialized Hospital, Ethiopia. Annals of clinical microbiology and antimicrobials 2013;12:17.

42. Hoque DM, Rahman M, Billah SM, et al. An assessment of the quality of care for children in eighteen randomly selected district and sub-district hospitals in Bangladesh. BMC pediatrics 2012;12:197.

46. Jawa R, Young D, Stothert J, Mercer D, Zakrison T, Freihaut F. A primer on mechanical ventilation in austere environments: Insights gained from post-earthquake haiti. Critical Care Congress of the Society of Critical Care Medicine 2011; San Diego, United States.

47. Jayashree M, Singhi S. Changing trends and predictors of outcome in patients with acute poisoning admitted to the intensive care. Journal of tropical pediatrics 2011;57:340-6.

48. Joshi S, Agarwal B, Malla G, Karmacharya B. Complete elimination of tetanus is still elusive in developing countries: a review of adult tetanus cases from referral hospital in Eastern Nepal. Kathmandu University medical journal 2007;5:378-81.

49. Kissoon N. Out of Africa--a mother's journey. Pediatric critical care medicine : a journal of the Society of Critical Care Medicine and the World Federation of Pediatric Intensive and Critical Care Societies 2011;12:73-9.

50. Kissoon N. Preventing intensive care admissions for sepsis in tropical Africa: PICASTA-food for thought. Pediatric critical care medicine : a journal of the Society of Critical Care Medicine and the World Federation of Pediatric Intensive and Critical Care Societies 2013;14:644-5.

51. Kpadonou GT, Alagnide E, Hounkpe PC, Atchade D, Labitan E. Rehabilitation of comatose patients in the intensive care unit. 25e Congres de Medecine Physique et de Readaptation; 2010; Marseille, France.

52. Lahpai JY, Khin Y. A clinical profile of children with acute kidney injury admitted to yangon children hospital. IPNA Congress; 2013; Shanghai, China.

54. Magutu V, Revathi G. Multi-drug resistant colonizers and pathogens in Critical Care Units: An observational study from routine laboratory data. 29th Congress of the International Academy of Pathology; 2012; Cape Town, South Africa.

56. Middleton J. Critical care in Cambodia. Nursing times 2012;108:35.

57. Muganyizi PS, Shagdara MS. Predictors of extra care among magnesium sulphate treated eclamptic patients at Muhimbili National Hospital, Tanzania. BMC pregnancy and childbirth 2011;11:41.

58. Mung'ayi V, Karuga R. Conformity to the surviving sepsis campaign international guidelines among physicians in a general intensive care unit in Nairobi. East African medical journal 2010;87:350-3.

65. Olsen OE, Ndeki S, Norheim OF. Complicated deliveries, critical care and quality in emergency obstetric care in Northern Tanzania. International journal of gynaecology and obstetrics: the official organ of the International Federation of Gynaecology and Obstetrics 2004;87:98-108.

67. Page AL, de Rekeneire N, Sayadi S, et al. Infections in children admitted with complicated severe acute malnutrition in Niger. PloS one 2013;8:e68699.

68. Paudel R, Panta OB, Paudel B, Paudel K, Pathak OK, Alurkar VM. Acute coronary syndrome in elderly - The difference compared with young in intensive care unit of a tertiary hospital in western Nepal. Journal of Clinical and Diagnostic Research 2009;3:1289-96.

69. Paudyal BP. Poisoning : pattern and profile of admitted cases in a hospital in central Nepal. JNMA; journal of the Nepal Medical Association 2005;44:92-6.

70. Penoyar T, Cohen H, Kibatala P, et al. Emergency and surgery services of primary hospitals in the United Republic of Tanzania. BMJ open 2012;2:e000369.

71. Phua J, Koh Y, Du B, et al. Management of severe sepsis in asia: A prospective observational study. 23rd Annual Congress of the European Society of Intensive Care Medicine; 2010; Barcelona, Spain.

72. **Pollach G**, **Anusa B**. TICASTA: Treating intensive care admissions for sepsis in tropical Africa. 6th International Congress "Sepsis and Multiorgan Dysfunction" - Weimar Sepsis Update 2013 - Consensus and Controversies 2013 2013; Weimar, Germany.

73. Pollach G, Anusa B. SICASTA: Supporting intensive care admissions for sepsis in tropical Africa. 6th International Congress "Sepsis and Multiorgan Dysfunction" - Weimar Sepsis Update 2013 - Consensus and Controversies 2013; Weimar, Germany.

74. Pollach G, Mindolo S. A population at risk for pediatric sepsis in Africa and its preoperative identification. 5th International Congress "Sepsis and Multiorgan Dysfunction"; 2011; Weimar, Germany.

75. Qusar MMAS, Morshed NM, Kader MA, Azad MAK, Uddin MA, Shaikh MAK. Psychiatric morbidity of suicide attempt patients requiring ICU intervention. Journal of Medicine (Bangladesh) 2010;11:7-11.

82. Shankar PR, Partha P, Dubey AK, Mishra P, Deshpande VY. Intensive care unit drug utilization in a teaching hospital in Nepal. Kathmandu University medical journal 2005;3:130-7.

84. Sherman L, Clement PT, Cherian MN, et al. Implementing Liberia's poverty reduction strategy: An assessment of emergency and essential surgical care. Archives of surgery 2011;146:35-9.

85. Shrestha R. Knowledge of central venous pressure (CVP) line among nursing personnel working at ICCU and dialysis dept of B.P. Koirala institute of health science, Nepal. 12th Asian Pacific Congress of Nephrology; 2010; Seoul, South Korea.

88. Sylla M, Folquet-Amorissani M, Oumar AA, et al. Neonatal morbidity and mortality in the pediatric intensive care service of Gabriel Toure Hospital. [French]. Louvain Medical 2009;128:141-4.

91. Thapa L, Paudel R, Rana PVS. Stroke mortality in intensive care unit (ICU): An experience from tertiary care neurological center, Nepal. World Stroke Congress; 2010; Seoul, South Korea.

96. Zoumenou E, Gbenou S, Assouto P, et al. Pediatric anesthesia in developing countries: experience in the two main university hospitals of Benin in West Africa. Paediatric anaesthesia 2010;20:741-7.

98. Ghimire M, Pahari B, Sharma SK, Thapa L, Das G, Das GC. Outcome of sepsis-associated acute kidney injury in an intensive care unit: An experience from a tertiary care center of central Nepal. Saudi journal of kidney diseases and transplantation : an official publication of the Saudi Center for Organ Transplantation, Saudi Arabia 2014;25:912-7.

99. Saidi H, Mutiso BK, Ogengo J. Mortality after road traffic crashes in a system with limited trauma data capability. Journal of trauma management & outcomes 2014;8:4.

100. Calland JF, Holland MC, Mwizerwa O, et al. Burn management in sub-Saharan Africa: opportunities for implementation of dedicated training and development of specialty centers. Burns : journal of the International Society for Burn Injuries 2014;40:157-63.

Wrong Country

2. Adudu OP, Ogunrin OA, Adudu OG. Morbidity and mortality patterns among neurological patients in the intensive care unit of a tertiary health facility. Annals of African medicine 2007;6:174-9.

3. Adudu P. Anesthetic equipment, facilities and services available for pediatric anesthesia in Nigeria. Nigerian journal of clinical practice 2012;15:75-9.

8. Angral R, Islam MS, Kundan S. Incidence of deep vein thrombosis and justification of chemoprophylaxis in Indian patients: a prospective study. Bangladesh Medical Research Council bulletin 2012;38:67-71.

24. Cohen J, Singer P, Kogan A, Hod M, Bar J. Course and outcome of obstetric patients in a general intensive care unit. Acta obstetricia et gynecologica Scandinavica 2000;79:846-50.

29. Ejiro BA, Edomwonyi NP. Audit of Intensive Care Unit (ICU) admissions from the operating room: Experience at the University of Benin Teaching Hospital, Benin City, Nigeria. Journal of Medicine and Biomedical Research 2012;11:9-17.

32. Galal NM. Pattern of intravenous immunoglobulins (IVIG) use in a pediatric intensive care facility in a resource limited setting. African health sciences 2013;13:261-5.

55. Markin A, Barbero R, Leow JJ, et al. A quantitative analysis of surgical capacity in Santa Cruz, Bolivia. The Journal of surgical research 2013;185:190-7.

62. Njall C, Adiogo D, Bita A, et al. [Bacterial ecology of nosocomial infection in intensive care unit of Laquintinie hospital Douala, Cameroon]. The Pan African medical journal 2013;14:140.

66. Omoigberale AI, Abiodun PO. Upsurge in neonatal tetanus in Benin City, Nigeria. East African medical journal 2005;82:98-102.

80. Sagaki P, Thanachartwet V, Desakorn V, et al. Clinical factors for severity of Plasmodium falciparum malaria in hospitalized adults in Thailand. PloS one 2013;8:e71503.

93. Ugochukwu O, Jerome A. An audit of intensive care unit admission in a pediatric cardio-thoracic population in Enugu, Nigeria. The Pan African medical journal 2010;6:10.

Wrong Topic

10. Assouto P, Tchaou B, Kangni N, et al. [Early outcome of digestive surgery in a tropical setting]. Medecine tropicale : revue du Corps de sante colonial 2009;69:477-9.

17. Brahmi N, Blel Y, Abidi N, et al. Methanol poisoning in Tunisia: report of 16 cases. Clinical toxicology 2007;45:717-20.

18. Budhathoki S, Poudel P, Shah D, et al. Clinical profile and outcome of children presenting with poisoning or intoxication: a hospital based study. Nepal Medical College journal : NMCJ 2009;11:170-5.

23. Cohen H, Penoyar T, Kibatala P, et al. A survey of emergency and surgical services in the United Republic of Tanzania. Annual Meeting of the Society for Academic Emergency Medicine; 2011; Boston, United States.

26. Dhungana SP, Shrestha SK, Kashyap AK, Piryani RM, Acharya GP. The etiology of fever in patients presented at KIST Medical College, Teaching Hospital, Lalitpur, Nepal. Nepal Medical College journal : NMCJ 2012;14:241-3.

33. Gnassingbe K, Simlawo K, Egbohou P, et al. Cardiac injury in child managed successfully in underprivileged hospital of Africa. World journal for pediatric & congenital heart surgery 2013;4:305-7.

35. Grosso P. A meta-analysis of 175 cases at emergency paediatric and surgical centre in goderich, Sierra Leone. 15th WFSA World Congress of Anaesthesiologists; 2012; Buenos Aires, Argentina.

36. Hanson J, Lam SW, Mohanty S, et al. Central venous catheter use in severe malaria: time to reconsider the World Health Organization guidelines? Malaria journal 2011;10:342.

37. Hanson JP, Lam SW, Mohanty S, et al. Fluid resuscitation of adults with severe falciparum malaria: effects on Acid-base status, renal function, and extravascular lung water. Critical care medicine 2013;41:972-81.

39. Hassan KA, Hasan MK, Chowdhury MG, Akhter H. Aspects of infection in intensive care unit--prevention and control. Mymensingh medical journal : MMJ 2010;19:474-6.

40. Holm M, Rueter J, Rudis M, Arendt C, Jensen N. Development of a pharmacy computerized inventory program (PCIP) in an emergency department/intensive care unit, outpatient care, and a pediatric hospital in Haiti. 2011 Annual Meeting of the American College of Clinical Pharmacy 2011; Pittsburgh, United States.

44. Iddriss A, Shivute N, Bickler S, et al. Emergency, anaesthetic and essential surgical capacity in the Gambia. Bulletin of the World Health Organization 2011;89:565-72.

45. Irimu G, Wamae A, Wasunna A, et al. Developing and introducing evidence based clinical practice guidelines for serious illness in Kenya. Archives of disease in childhood 2008;93:799-804.

53. Lakhey S, Shrestha R, Thapa S, Tuladhar S. Scenario of acute respiratory distress syndrome in a tertiary care center. JNMA; journal of the Nepal Medical Association 2010;49:129-32.

59. Nahar A, Anwar S, Miah MRA. Association of biofilm formation with antimicrobial resistance among the Acinetobacter species in a tertiary care hospital in Bangladesh. Journal of Medicine (Bangladesh) 2013;14:28-32.

60. Nayak SK, Sherchan M, Dutta Poudel S, et al. Assessing placement of nasoduodenal tube and its usefulness in maintaining nutrition in critically ill patients. Nepal Medical College journal : NMCJ 2008;10:249-53.

61. Ndugulile F, Jureen R, Harthug S, Urassa W, Langeland N. Extended spectrum beta-lactamases among Gram-negative bacteria of nosocomial origin from an intensive care unit of a tertiary health facility in Tanzania. BMC infectious diseases 2005;5:86.

63. Okafor UV, Efetie ER, Amucheazi A. Risk factors for maternal deaths in unplanned obstetric admissions to the intensive care unit-lessons for sub-Saharan Africa. African journal of reproductive health 2011;15:51-4.

64. Okee MS, Joloba ML, Okello M, et al. Prevalence of virulence determinants in Staphylococcus epidermidis from ICU patients in Kampala, Uganda. Journal of infection in developing countries 2012;6:242-50.

76. Ralston ME, Day LT, Slusher TM, Musa NL, Doss HS. Global paediatric advanced life support: improving child survival in limited-resource settings. Lancet 2013;381:256-65.

77. Rampanjato R, Claude N, Paulin B. Descriptive study of a capacity assessment tool to emergency medical care service delivery at the district health level. 15th WFSA World Congress of Anaesthesiologists; 2012; Buenos Aires, Argentina.

78. Randrianirina F, Vaillant L, Ramarokoto CE, et al. Antimicrobial resistance in pathogens causing nosocomial infections in surgery and intensive care units of two hospitals in Antananarivo, Madagascar. Journal of infection in developing countries 2010;4:74-82.

81. Saka B, Barro-Traore F, Atadokpede FA, et al. Stevens-Johnson syndrome and toxic epidermal necrolysis in sub-Saharan Africa: A multicentric study in four countries. International Journal of Dermatology 2013;52:575-9.

86. Shrestha S, Karki U. Indications of admission and outcome in a newly established neonatal intensive care unit in a developing country (Nepal). Nepal Medical College journal : NMCJ 2012;14:64-7.

87. Singh PM, Shrestha DM, Tajhya RB, Shakya S. Delirium at Nepal Medical College Teaching Hospital: reason for referral and subtypes. Nepal Medical College journal : NMCJ 2009;11:28-30.

89. Talha KA, Hasan Z, Selina F, Palash MI. Organisms associated with ventilator associated pneumonia in intensive care unit. Mymensingh medical journal : MMJ 2009;18:S93-7.

90. Thaler A, Dunlevy H, Cohn J, Speck R, O'Brien M, McCunn M. A basic needs assessment of Kenyan health care practitioners' training and ability in providing resuscitation management for patients in Mbagathi Hospital, Nairobi. Journal of clinical anesthesia 2013;25:388-92.

92. Tripathi M, Pandey M, Nepal B, Rai H, Bhattarai B. Evaluation of lung infiltration score to predict postural hypoxemia in ventilated acute respiratory distress syndrome patients and the lateralization of skin pressure sore. Indian journal of medical sciences 2009;63:392-401.

97. Tariq TM. Occurrence of Extended-Spectrum b-Lactamase Producers Among Enterobacteriaceae in a Paediatric Tertiary Care Facility in Kabul. Journal of the College of Physicians and Surgeons--Pakistan : JCPSP 2014;24:530-1.

101. Elkheir N, Sharma A, Cherian M, et al. A cross-sectional survey of essential surgical capacity in Somalia. BMJ open 2014;4:e004360.

Military

9. Arul GS, Reynolds J, DiRusso S, et al. Paediatric admissions to the British military hospital at Camp Bastion, Afghanistan. Annals of the Royal College of Surgeons of England 2012;94:52-7.

38. Harris CC, McNicholas JJ. Paediatric intensive care in the field hospital. Journal of the Royal Army Medical Corps 2009;155:157-9.

41. Hopfner R, Tran T, Khan N, Nares M. Practicing pediatric critical care medicine after a natural disaster: Lessons learned from the earthquake in haiti. Critical Care Congress of the Society of Critical Care Medicine 2011; San Diego, United States.

43. Hotz GA, Moyenda ZB, Bitar J, et al. Developing a trauma critical care and rehab hospital in Haiti: a year after the earthquake. American journal of disaster medicine 2012;7:273-9.

94. von Saint Andre-von Arnim A, Brogan TV, Hertzig J, et al. Intensive care for infants and children in Haiti in April 2010. Pediatric critical care medicine : a journal of the Society of Critical Care Medicine and the World Federation of Pediatric Intensive and Critical Care Societies 2011;12:393-7.

95. Wilson KL, Schenarts PJ, Bacchetta MD, Rai PR, Nakayama DK. Pediatric trauma experience in a combat support hospital in Eastern Afghanistan over 10 months, 2010 to 2011. American Surgeon 2013;79:257-60.

102. Belenkiy SM, Buel AR, Cannon JW, et al. Acute respiratory distress syndrome in wartime military burns: application of the Berlin criteria. The journal of trauma and acute care surgery 2014;76:821-7.

Duplicate

11. Baelani I, Jochberger S, Laimer T, et al. Availability of critical care resources to treat patients with severe sepsis or septic shock in Africa: a self-reported, continent-wide survey of anaesthesia providers. Critical care 2011;15:R10.

27. Dhungel S, Bista S. High blood glucose level and increased risk of mortality in critically ill patients. Nepal Medical College journal : NMCJ 2007;9:44-5.

43. Hotz GA, Moyenda ZB, Bitar J, et al. Developing a trauma critical care and rehab hospital in Haiti: a year after the earthquake. American journal of disaster medicine 2012;7:273-9.

79. Riviello ED, Letchford S, Achieng L, Newton MW. Critical care in resource-poor settings: lessons learned and future directions. Critical care medicine 2011;39:860-7.

83. Shavadia J, Yonga G, Otieno H. A prospective review of acute coronary syndromes in an urban hospital in sub-Saharan Africa. Cardiovascular journal of Africa 2012;23:318-21.

94. von Saint Andre-von Arnim A, Brogan TV, Hertzig J, et al. Intensive care for infants and children in Haiti in April 2010. Pediatric critical care medicine : a journal of the Society of Critical Care Medicine and the World Federation of Pediatric Intensive and Critical Care Societies 2011;12:393-7.

103. Basnet S, Adhikari N, Avila M, Kache S. Establishing intensive care units in Nepal. Society of Critical Care Medicine; 2009; Miami, USA.

104. Basnet S, Adhikari N, Koirala J. Challenges in setting up pediatric and neonatal intensive care units in a resource-limited country. Pediatrics 2011;128:e986-92.

105. Khun PA, Seng S, Emary K, et al. Surveillance of healthcare-associated infection at Angkor Hospital for Children, Siem Reap, Cambodia. International Journal of Infectious Diseases 2012:e375.

106. Muteya MM, Kabey AK, Lubanga TM, Tshamba HM, Nkoy AM. Prognosis of tetanus patients in the intensive care unit of Provincial Hospital Jason Sendwe, Lubumbashi, DR Congo. The Pan African medical journal 2013;14:93.

107. Baker T, Lugazia E, Eriksen J, Konrad D. Emergency and critical care services in Tanzania. ESICM LIVES; 2011; Berlin, Germany.

108. Phua J, Koh Y, Du B, et al. Management of severe sepsis in asia: A prospective observational study. ESICM; 2010; Barccelona, Spain.
